# Cardiac-Oxidized Antigens Are Targets of Immune Recognition by Antibodies and Potential Molecular Determinants in Chagas Disease Pathogenesis

**DOI:** 10.1371/journal.pone.0028449

**Published:** 2012-01-04

**Authors:** Monisha Dhiman, Maria Paola Zago, Sonia Nunez, Alejandro Amoroso, Hugo Rementeria, Pierre Dousset, Federico Nunez Burgos, Nisha Jain Garg

**Affiliations:** 1 Department of Microbiology and Immunology, Center for Tropical Diseases, Institute for Human Infections and Immunity, University of Texas Medical Branch, Galveston, Texas, United States of America; 2 Facultad de Ciencias de la Salud, Instituto de Patología Experimental, Universidad Nacional de Salta, Salta, Argentina; 3 Hospital Público de Gestión Descentralizada San Bernardo, Salta, Argentina; 4 Servicio de Cirugia Cardiovascular, Hospital San Bernardo, Salta, Argentina; 5 Center for Tropical Diseases, Institute for Human Infections and Immunity, University of Texas Medical Branch, Galveston, Texas, United States of America; University of South Alabama, United States of America

## Abstract

*Trypanosoma cruzi* elicits reactive oxygen species (ROS) of inflammatory and mitochondrial origin in infected hosts. In this study, we examined ROS-induced oxidative modifications in the heart and determined whether the resultant oxidized cardiac proteins are targets of immune response and of pathological significance in Chagas disease. Heart biopsies from chagasic mice, rats and human patients exhibited, when compared to those from normal controls, a substantial increase in protein 4-hydroxynonenal (4-HNE), malondialdehyde (MDA), carbonyl, and 3-nitrotyrosine (3-NT) adducts. To evaluate whether oxidized proteins gain antigenic properties, heart homogenates or isolated cardiomyocytes were oxidized *in vitro* and one- or two-dimensional gel electrophoresis (2D-GE)/Western blotting (WB) was performed to investigate the proteomic oxidative changes and recognition of oxidized proteins by sera antibodies in chagasic rodents (mice, rats) and human patients. Human cardiomyocytes exhibited LD_50_ sensitivity to 30 µM 4-HNE and 100 µM H_2_O_2_ at 6 h and 12 h, respectively. In *vitro* oxidation with 4-HNE or H_2_O_2_ resulted in a substantial increase in 4-HNE- and carbonyl-modified proteins that correlated with increased recognition of cardiac (cardiomyocytes) proteins by sera antibodies of chagasic rodents and human patients. 2D-GE/Western blotting followed by MALDI-TOF-MS/MS analysis to identify cardiac proteins that were oxidized and recognized by human chagasic sera yielded 82 unique proteins. We validated the 2D-GE results by enzyme-linked immunosorbent assay (ELISA) and WB and demonstrated that oxidation of recombinant titin enhanced its immunogenicity and recognition by sera antibodies from chagasic hosts (rats and humans). Treatment of infected rats with phenyl-α-tert-butyl nitrone (PBN, antioxidant) resulted in normalized immune detection of cardiac proteins associated with control of cardiac pathology and preservation of heart contractile function in chagasic rats. We conclude that ROS-induced, cardiac-oxidized antigens are targets of immune recognition by antibodies and molecular determinants for pathogenesis during Chagas disease.

## Introduction

*Trypanosoma cruzi* is the etiologic agent of Chagas disease. According to World Health Organization estimates, the overall prevalence of human *T. cruzi* infection is at ∼16–18 million cases, and ∼120 million people are at risk of infection [1]. Upon exposure to *T. cruzi*, hosts elicit strong B and T cell-mediated immune responses, resulting in control of acute parasitemia (reviewed in [2], [3]). Approximately 30–40% of the infected individuals, several years after initial exposure, develop clinical symptoms of irreversible cardiomyopathy, including cardiomegaly, ventricular dilation and arrhythmia, and heart failure.

Immune-mediated cytotoxic reactions are the source of reactive oxygen species (ROS, e.g. O_2_^•−^, H_2_O_2_, ^•^OH) during acute *T. cruzi* infection [2], [4]. Macrophages and neutrophils present the first line of defense and contribute to parasite control through respiratory burst [5], [6] supported by activation of NADPH oxidase (NOX) [7], myeloperoxidase (MPO) [8] and inducible nitric oxide synthase (iNOS) [9]. In addition, mitochondrial dysfunction of the electron transport chain resulting in increased leakage of electrons to molecular oxygen serves as a main source of ROS in the heart [10]. ROS and secondary by-products of oxidative stress induce many different types of protein modifications [11]. For example, ROS react with cysteine-, histidine- and lysine-residues of proteins to form 4-HNE [12]. In addition, protein-derived aldehydes and ketones are produced by direct oxidation of arginine, lysine, proline, or threonine residues [13], and collectively termed protein carbonyls [12]. ROS modification of membrane polyunsaturated lipids results in the formation of MDA. Tyrosine residues on proteins are susceptible to attack by peroxynitrite and result in stable polypeptide-bound 3-nitrotyrosine (3-NT) residues [14]. These oxidative modifications of proteins, if not appropriately prevented, can have cytotoxic effects on the host.

It was recently shown that oxidative stress-induced protein modifications occur in the myocardium of *T. cruzi*-infected experimental animals [15], [16], [17] and peripheral blood of chagasic animals [18] and human patients [19], [20]. The enhanced peripheral oxidative stress was associated with non-responsive glutathione antioxidant defense [19], [21] and increased MPO activity and ROS production in seropositive chagasic patients [8], [19], [22]. The extent of myocardial protein oxidation was pronounced in acute conditions [18] and persisted during the chronic disease stage. Further, treatment of infected rodents with an antioxidant (with or without anti-parasite drug) was effective in controlling the cardiac oxidative pathology [23] and the loss of left ventricle (LV) function in the chronic stage [24], thus, establishing the pathological significance of oxidative overload in Chagas disease.

In this study, we investigated whether oxidative modification of cardiac proteins occurs in human patients and results in the formation of new epitopes that, if recognized by B cell-mediated immune responses, would lead to an immune recognition of the host and be of pathological importance in cardiac damage during Chagas disease. We employed an *in vitro* system of oxidative modification of human cardiomyocytes or heart homogenates with oxidants and utilized 2D-GE/Western blotting and mass spectrometry approaches to identify the cardiac oxidized proteins that were targets of antibody responses in chagasic human patients. Further, we used an experimental model to determine whether the beneficial effects of treatment with PBN antioxidant in controlling myocardial oxidative stress and the resultant LV dysfunction [24] were associated with control of cardiac oxidized antigen formation and self-directed antibody response in chagasic animals. Our findings of a number of cardiac proteins that were immunogenic due to oxidative stress-induced modifications in chagasic patients and rodents and normalized by PBN treatment in infected rodents provide clues to the role of cardiac-oxidized antigens in eliciting self-targeted immune responses during Chagas disease.

## Materials and Methods

### Animal and parasites

Animal experiments were performed according to the National Institutes of Health Guide for Care and Use of Experimental Animals and approved by the University of Texas Medical Branch Animal Care and Use Committee (ID: 0805029). *T. cruzi* trypomastigotes (SylvioX10/4 strain) were propagated in C2C12 cells (ATCC, CRL-1772). C3H/HeN mice (4- to 5-weeks-old, Harlan Labs) were infected with *T. cruzi* (10,000 parasites/mouse, intraperitoneally) and harvested at day 25 and 150 post-infection (dpi) corresponding to acute and chronic stages of infection and disease development, respectively. Sprague Dawley rats (4- to 5-weeks-old, Harlan Labs) were infected with *T. cruz*i (50,000-trypomastigotes/rat, intraperitoneally). Rats were given 1.3 mM PBN (beginning day 0, throughout the course of infection) in drinking water. The rat plasma samples were obtained at days 40 and 180 post-infection, corresponding, respectively, to acute and chronic stages of infection and disease development.

### Human samples

All procedures for human sample collection were approved by the institutional review boards at the University of Texas Medical Branch (ID: 04–257) and the Universidad Nacional de Salta, Argentina. Written informed consent was obtained from all individuals before their enrollment in the study. Blood samples were collected with heparin or without anticoagulant to obtain plasma and serum, respectively. Trained medical personnel at the Central Laboratory of San Bernardo Hospital (Salta, Argentina) conducted blood sampling by venipuncture. Three different serology tests were used to test sera samples for *T. cruzi*-specific antibodies. Those positive by at least two tests were identified as seropositive. Data included medical history, physical examination and subjective complaint of frequency and severity of exertional dyspnea, along with results of electrocardiography (12-leads at rest and 3-leads with exercise) to reveal cardiac rhythm and conduction abnormalities, transthoracic echocardiogram to obtain objective information regarding the left ventricular (LV) contractile function, and chest X-ray to assess cardiomegaly (cardio-thoracic ratio >0.5). The severity of exertional dyspnea was graded according to the New York Heart Association Classification (NYHA) [25]. Based on these criteria, seropositive chagasic patients exhibiting no echocardiography abnormalities, preserved systolic function (ejection fraction (EF) ≥55%), no LV dilatations, but with negligible-to-minor EKG alterations were graded as CD0 or CD1, respectively. Seropositive patients were graded CD2 with mild-to-moderate systolic dysfunction (EF: 40–54%) and/or LV dilatation, and CD3 with severe systolic dysfunction (LV end diastolic diameter ≥57 mm, EF ≤40%). Seronegative cardiomyopathy patients of other etiologies (OCM) were categorized similar to the chagasic patients. Normal, seronegative and healthy individuals exhibiting no history or clinical symptoms of cardiac disease were used as controls. Cardiac biopsies were obtained from chagasic patients undergoing correctional surgical intervention for clinical purposes at the San Bernardo Hospital (Salta, Argentina). Normal cardiac biopsies were obtained from the National Disease Research Interchange (NDRI) tissue bank.

### Immunohistochemistry

Heart tissue sections were fixed in formalin and embedded in paraffin. Tissue sections (5-µm) were subjected to immunostaining for 2 h at room temperature with antibody against 4-HNE (1∶500, Alpha Diagnostics) to visualize aldehydic adducts, and dinitrophenylhydrazine (DNPH, 1∶200, Sigma) to detect DNP-derivatized carbonyl proteins. Slides were then incubated for 30 min each with biotinylated IgG (1∶200) and streptavidin-conjugated horseradish peroxidase (HRP, 1∶200, Dako), and color was developed using 3,3′ diaminobenzidine substrate (Sigma). Each tissue section (3 sections/tissue sample) was analyzed for >10-microscopic fields and scored for adducts as a percentage of total histological field quantified by using computerized Simple PCI Software version 6.0 (Compix). In some experiments, slides were incubated with rhodamine-conjugated secondary antibody, and fluorescence was visualized on an Olympus BX-15 microscope, and images captured by using a mounted digital camera.

### Cell viability assay

Cardiomyocytes (10^6^/ml) were *in vitro* incubated for 0, 2, 6, 12, and 24 h with 0–40 µM 4-HNE (Cayman) or 0–200 µM H_2_O_2_ (Sigma). Cells were then incubated with 3-(4,5-dimethylthiazol-2-yl)-2,5-diphenyltetrazolium bromide (MTT, 5 mg/ml) for 3 h. The reduction of MTT by mitochondrial dehydrogenases of the live cells resulted in the formation of purple formazan crystals that were solubilized in dimethylsulfoxide and absorbance measured at 540 nm by using a spectrophotometer. ROS efficiency was determined by the lowest concentration of ROS that elicited 50% cell death in minimal time (Percent viability: [mean OD of samples/mean OD of controls]×100) [26].

### Tissue and cell homogenates

Heart tissue section (tissue: buffer ratio, 1∶10 w/v) or cardiomyocytes (10^6^ cells/ml) were washed with ice-cold Tris-buffered saline and homogenized in lysis buffer [15]. Homogenates (1 mg protein/ml) were *in vitro* oxidized with 30 µM 4-HNE or 100 µM H_2_O_2_ for 6 h and 12 h, respectively, centrifuged at 3,000 g, 4°C for 10-min and the resulting supernatant aliquots stored at −80°C.

### Two-dimensional gel electrophoresis

The 11-cm immobilized pH gradient (IPG) strips (pH: 3–10, Bio-Rad) were rehydrated at 50 V for 12 h with 250-µl rehydration buffer (1 M thiourea, 8 M urea, 2% CHAPS, 1% dithiothreitol, and 0.2% ampholytes) containing 100-µg protein samples and 0.002% of bromophenol blue. Isoelectric focusing was performed at 500 V for 1 h, 1000 V for 1 h, 8000 V for 2 h, and then at 8000 V for a total of 50,000 V [17]. The IPG strips were suspended in equilibration buffer (50 mM Tris-HCl, pH 6.8, 6 M urea, 20% glycerol) and sequentially incubated for 15 min each in the presence of 2% dithiothreitol/2% SDS (reducing conditions) and 2.5% iodoacetamide/2% SDS (alkylating conditions). Equilibrated IPG strips were subjected to second-dimension electrophoresis by using 8–10% linear gradient precast Tris-HCl gels (Bio-Rad) on a PROTEAN plus Dodeca Cell System at 75 V for 1 h and then at 120 V until the dye front reached the bottom of the gel. Gels were fixed in 10% methanol/7% acetic acid, stained with SYPRO Ruby (BioRad), destained in 10% ethanol/7% acetic acid, and imaged by using a high-resolution ProXPRESS Proteomic Imaging System (Perkin Elmer).

### Protein oxidative modifications

Tissue or cardiomyocyte homogenates (normal and *in vitro* oxidized) were resolved by 1-dimension (10 µg protein/well) or 2-dimension (100 µg protein) gel electrophoresis and proteins transferred to PVDF membranes by using a wet, vertical Criterion Blotter (Bio-Rad). Membranes were blocked for 1 h with 5% nonfat dry milk (NFDM, BioRad) in 50 mM Tris-HCl (pH 7.4) containing 150 mM NaCl and 0.05% Tween-20 (TBST). Membranes were incubated overnight at 4°C with antibodies against 4-HNE (1∶2000, Alpha Diagnostics), MDA (1∶3000, Biodesign), 3-NT (1∶2000, Cell Signaling), or DNP (1∶1000, Invitrogen), and washed three times. All antibody dilutions were made in 5% NFDM-TBST. Membranes were then incubated with appropriate HRP-conjugated secondary antibody for 1 h, and signal was developed by using an ECL Plus system (GE-Healthcare). Membranes were stained with Coomassie blue G250 (Bio-Rad) to confirm an equal loading of samples. Images were visualized and digitized, and densitometric analysis was performed by using a FluorChem 8800 Image Analyzer System (Alpha Innotech).

For the identification of protein carbonyls by 2D-GE, IPG strips were loaded with protein samples and subjected to 1^st^ dimension isoelectric focusing, as above. IPG strips were then incubated with 1 ml of 3% SDS and 5 mM DNPH/10% trifluoroacetic acid for 25 min. After neutralization with 1.5 ml of 2 M Tris, 30% glycerol, DNP-derivatized protein samples in IPG strips were subjected to 2^nd^ dimension electrophoresis by using 8–10% gradient precast gels (BioRad), and transferred to PVDF membranes. Membranes were hybridized with rabbit anti-DNP antibody (1∶300, Invitrogen) and HRP-conjugated goat anti-rabbit secondary antibody (1∶1000, Southern Biotech), and signal detected as above.

### Immune recognition of cardiac proteins by chagasic sera

Protein homogenates of cardiomyocytes or heart tissue (normal and *in vitro* oxidized) were resolved by 1D- or 2D-GE, and transferred to PVDF membranes, as above. Membranes were probed with sera from normal or chagasic rats, mice or human patients (1∶100 dilution) followed by HRP-conjugated secondary antibody (1∶5000, BioRad), and signal was detected by an ECL plus chemiluminiscence detection system (GE-Healthcare).

### Image analysis

2D gels (n = 4/group) or Western blots (n = 4/group) were digitalized on a ProXPRESS Proteomic Imaging System (Perkin Elmer), and the images were analyzed on Progenesis SameSpotst™ software 2.0 (NonLinear Dynamics). Normalized spot volumes, i.e., the volume of each spot over the volume of all spots in the gel, were used to compare the different groups, and candidates were identified as protein spots that changed at least 2-fold versus their specific control. Statistical significance was assessed by a two-tailed Student's *t*-test and analysis of variance test (ANOVA) [27], and the *p* values of <0.05 were considered significant for comparison between control and experimental data.

### Mass spectrometry and protein identification

Selected spots (1-mm) were excised from the gels and submitted to trypsin proteolysis as described [17]. In brief, gel spots were incubated at 37°C for 30 min in 50 mM NH_4_HCO_3_, dehydrated twice for 5 min each in 100-µl acetonitrile and dried, and then, in-gel proteins were digested at 37°C for 6 h with 10 µl of trypsin solution (1% trypsin in 25 mM ammonium bicarbonate). After digestion, 1 µl of peptide mixture was directly spotted onto a MALDI-TOF-MS/MS target plate with 1 µl of alpha-cyano-4-hydroxycinnamic acid matrix solution (5 mg/ml in 50% acetonitrile). Peptides were analyzed by using a MALDI-TOF/TOF ABI 4800 Proteomics Analyzer (Applied Biosystems). The Applied Biosystems software package included the 4000 Series Explorer (v. 3.6 RC1) with Oracle Database Schema Version (v. 3.19.0) and Data Version (3.80.0) to acquire and analyze MS and MS/MS spectral data. The instrument was operated in a positive ion reflectron mode with the focus mass set at 1700 Da (mass range: 850–3000 Da). For MS data, 1000–2000 laser shots were acquired and averaged from each protein spot. Automatic external calibration was performed by using a peptide mixture with the reference masses 904.468, 1296.685, 1570.677, and 2465.199. MALDI MS/MS was performed on several (5–10) abundant ions from each protein spot. A 1-kV positive ion MS/MS method was used to acquire data under post-source decay (PSD) conditions. The instrument precursor selection window was +/− 3 Da. Automatic external calibration was performed by using reference fragment masses 175.120, 480.257, 684.347, 1056.475, and 1441.635 (from precursor mass 1570.700).

Applied Biosystems GPS Explorer™ (v. 3.6) software was used in conjunction with MASCOT to search the respective protein database by using both MS and MS/MS spectral data for protein identification. Protein match probabilities were determined by using expectation values and/or MASCOT protein scores. The MS peak filtering included the following parameters: a mass range of 800 Da to 3000 Da, minimum S/N filter = 10, mass exclusion list tolerance = 0.5 Da and mass exclusion list for trypsin and keratin-containing compounds included masses 842.51, 870.45, 1045.56, 1179.60, 1277.71, 1475.79, and 2211.1. The MS/MS peak filtering included the following parameters: minimum S/N filter = 10, maximum missed cleavages = 1, fixed modification of carbamidomethyl (C), variable modifications due to oxidation (M), precursor tolerance = 0.2 Da, MS/MS fragment tolerance = 0.3 Da, mass = monoisotopic, and peptide charges = +1. The significance of a protein match, based on the peptide mass fingerprint (PMF) in the MS and the MS/MS data from several precursor ions, is presented as expectation values (*p*<0.001).

### Enzyme-linked immunosorbent assay

Human recombinant titin (0.1 mg/ml, Novus Biologicals) was sonicated three times for 30 sec each at 4°C, and then *in vitro* oxidized with 4-HNE (30 µM) or H_2_O_2_ (100 µM) for 1 h at 37°C. A soluble lysate of culture-derived *T. cruzi* (70% trypomastigotes, 30% amastigotes) was prepared as previously described [28], and used as a control. Flexible U bottom (96-well) polyvinyl chloride plates (BD Biosciences) were coated overnight at 4°C with 0.5 µg/100 µl/well of titin. Plates were blocked for 2 h at 37°C with 200-µl/well of 1% non-fat dry milk in PBS. After washing with PBS-0.05% Tween-20 (PBST) and PBS, plates were sequentially incubated at room temperature for 2 h with test sera (1∶100) and 30 min with HRP-labeled isotype-matched IgG (1∶5000, BioRad), and color was developed with 100 µl/well Sure Blue 3,3′,5,5′-tetramethylbenzidine substrate (Kirkegaard & Perry Labs). After stopping the reaction with 2N sulfuric acid, absorbance was monitored at 450 nm by using a SpectraMax 190 microplate reader (Molecular Devices).

### Data analysis

Data (mean ± SD) were derived from at least triplicate observations per sample (>4 samples/group). Normally distributed data were analyzed by student's t-test. The level of significance is presented by *^,#^ *p*<0.01, **^,##^ *p*<0.001 (*normal versus infected, ^#^infected/untreated versus infected/PBN-treated).

## Results

### Oxidative adducts are enhanced in experimental and human myocardium during Chagas disease

**Figure 1:**
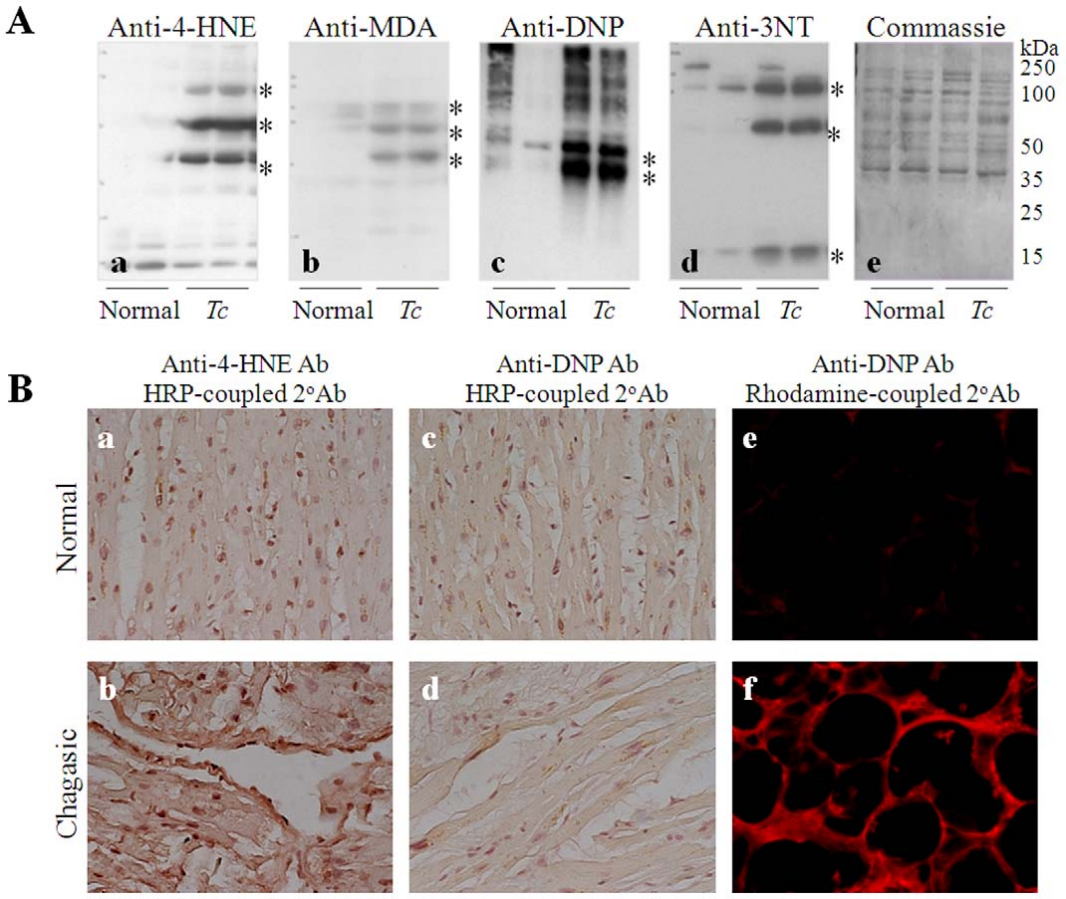
Oxidative adducts are enhanced in experimental and human myocardium during Chagas disease. (**A**) Sprague-Dawley rats (or C3H/HeN mice) were infected with *T. cruzi,* and cells were harvested at day 40 (acute stage) and 180 (chronic stage) post-infection. Heart homogenates were resolved on 10% acrylamide gels, and Western blotting was performed with specific antibodies to detect 4 hydroxynonenal (4-HNE, *panel a*), malondialdehyde (MDA, *panel b*), dinitrophenyl (DNP)-derivatized carbonyl (*panel c*), and 3-nitrotyrosine (3NT, *panel d*) adducts. Coomassie blue staining of membranes (*panel e*) confirmed the equal loading of samples. (**B**) Cryostat sections of human cardiac biopsies (5-µm) from normal healthy donors (*panels a, c, e*) and chagasic patients (*panels b, d, f*) were submitted to immunohistochemistry as described in Materials and Methods. Shown are representative images of immunostaining with anti-4-HNE antibodies (*panels a, b*). Tissue sections were incubated with DNPH to derivatize carbonyl proteins, and immunostaining was performed with anti-DNP antibody (*panels c–f*). HRP-conjugated (*panels a–d*) and rhodamine-conjugated (*panels e, f*) secondary antibodies were utilized to capture the color (brown) or fluorescence signal, respectively.

Many different types of protein modifications, e.g., aldehydic adducts (4-HNE and MDA), carbonyl derivatives, and 3-NT can be induced by ROS and secondarily by products of oxidative stress. We performed 1D-GE/WB and immunohistochemistry to detect oxidative adducts in cardiac biopsies of infected rodents and human patients (Fig. 1). Densitometry analysis of selected bands (marked by asterisk) showed 5-fold, 2-fold, 3-fold and 2-fold increase in the myocardial levels of 4-HNE, MDA, DNP-derivatized carbonyl, and 3-NT adducts, respectively, in chagasic rats (Fig. 1A.a–d) and mice (data not shown). The increased level of protein oxidative adducts in the heart was observed both during the acute stage of high parasitemia (Fig. 1A.a–d) and chronic stage of disease development (data not shown) in infected rodents. Coomassie blue staining of the membranes confirmed equal loading of the samples (Fig. 1A.e).

Cardiac tissue biopsies from normal healthy donors and chagasic human patients were subjected to immunohistochemistry to detect oxidative adducts (Fig. 1B). Our data showed the myocardial level of 4-HNE (Fig. 1B.b, score 3) and carbonyl (Fig. 1B.d & f, score 2 and 3) protein modifications were significantly increased in chagasic patients in comparison to that noted in healthy controls (Fig. 1B.a,c,e). In comparison, a marginal increase in myocardial level of carbonyl and 4-HNE adducts was observed in cardiomyopathy patients of other etiologies (data not shown). Together, these data suggested to us that cardiac proteins are targets of oxidative modifications during infection by *T. cruzi* and Chagas disease development in both experimental animals and human patients.

### Oxidative stress-induced changes affected cardiomyocytes viability

**Figure 2:**
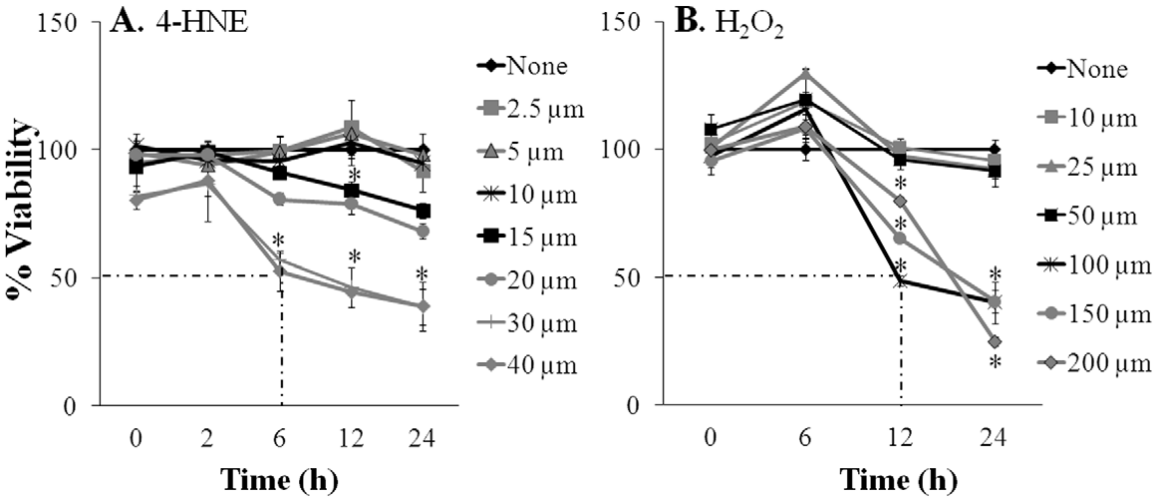
Oxidative stress affects cardiomyocyte viability. Human cardiomyocytes were incubated with (**A**) 4-hydroxynonenal (0–40 µM) or (**B**) H_2_O_2_ (0–200 µM) for 0, 2, 6, 12, and 24 h. An MTT assay was performed to examine cell viability (*p<0.05).

Next, we determined if ROS-induced oxidative modifications affect cell viability. Cardiomyocytes (mouse and human, 10^6^/ml) were treated *in vitro* with 4-HNE (0–40 µM) or H_2_O_2_ (0–200 µM) for 0–24 h and an MTT assay was performed to assess cell viability. Our data showed that human cardiomyocytes were resistant to 5 µM 4-HNE and 50 µM H_2_O_2_ for at least 12 h and exhibited only a 12% loss in viability at 24 h (Fig. 2A&B), possibly indicative of an endogenous antioxidant activity. Human cardiomyocytes exhibited a linear decline in viability in response to 20–40 µM 4-HNE (Fig. 2A) and 50–200 µM H_2_O_2_ (Fig. 2B), the slope of decline in viability being steeper with increasing concentration of the oxidant. The minimal LD_50_ value for 4-HNE and H_2_O_2_ was 30 µM and 100 µM, resulting in a 50 percent cell death at 6 h and 12 h post-treatment, respectively. Similar results were obtained when murine cardiomyocytes were treated with 4-HNE or H_2_O_2_ (data not shown). These data suggested that oxidative adducts affect the viability of cardiomyocytes in a dose- and time-dependent manner. We used cells or tissue lysates (10^6^ cardiomyocyte equivalent protein concentration/ml) treated with 30 µM 4-HNE and 100 µM H_2_O_2_ for 6 h and 12 h, respectively, for all additional studies.

### Identification of cardiac proteins susceptible to oxidative stress

**Figure 3:**
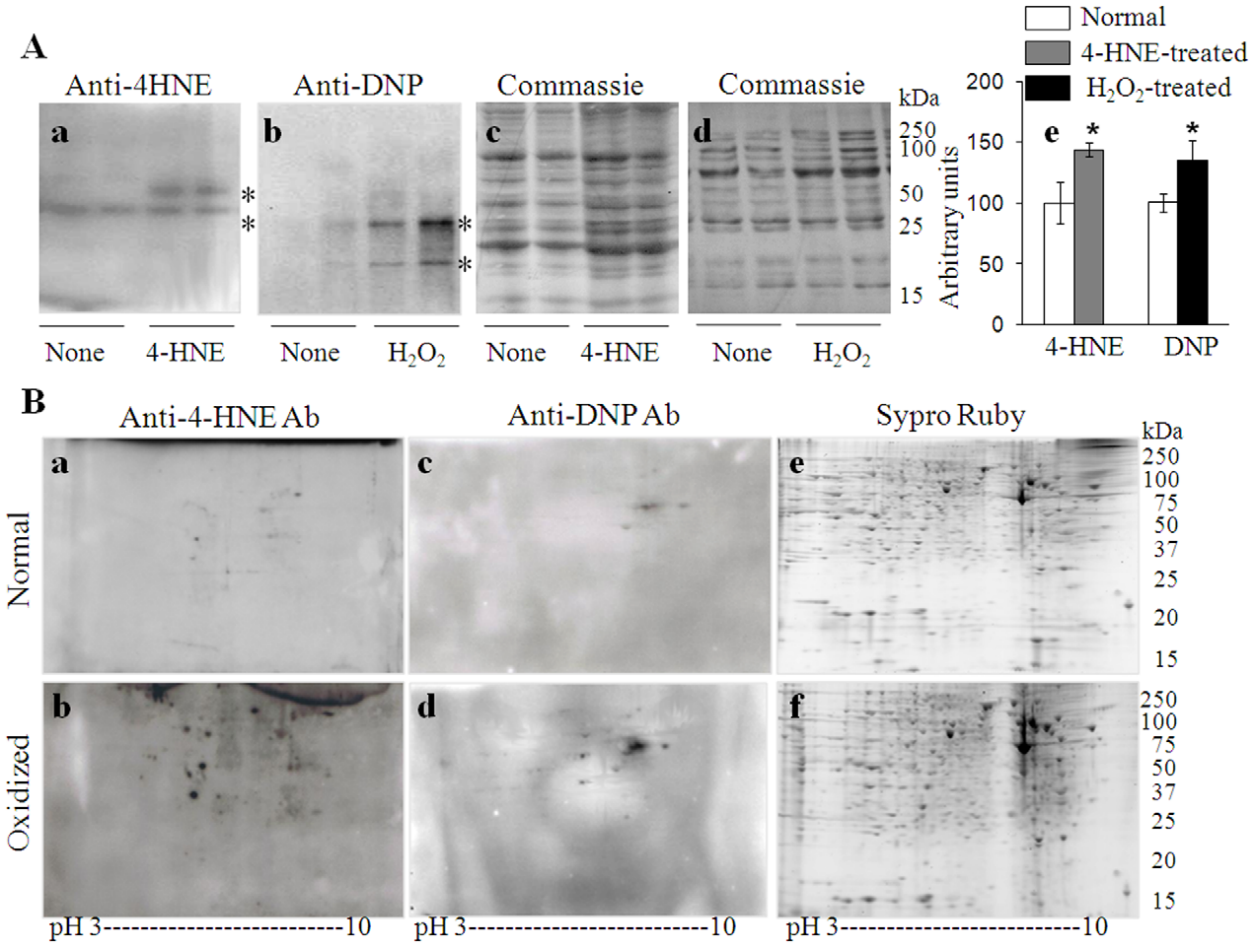
Identification of cardiomyocyte proteins susceptible to oxidative stress. (**A**) Rat heart homogenates were treated *in vitro* with 30 µM 4-HNE or 100 µM H_2_O_2_, and Western blotting was performed to detect 4-HNE (*panel a*) and DNP-derivatized carbonyl (*panel b*) proteins. Coomassie blue staining of membranes (*panels c, d*) confirmed the equal loading of samples. Densitometry analysis of oxidized proteins (marked with asterisk in *panels a & b*) is shown in *panel e* (**B**) Human cardiomyocyte homogenates (1 mg protein/ml) were treated for 6 h with 30 µM 4-HNE or 12 h with 100 µM H_2_O_2_. Normal (*panels a, c, e*), 4-HNE-treated (*panel b*) and H_2_O_2_-treated (panels *d, f*) cell homogenates were subjected to 1^st^ dimension isoelectric focusing on 11-cm, linear pH 3–10 immobilized pH gradient (IPG) strips. Strips were incubated with DNPH to derivatize carbonyl proteins, and 2^nd^ dimension separation was carried out on 8–10% gradient gels. Membranes were probed with anti-4-HNE (*panels a, b*) and anti-DNP (*panels c, d*) antibodies. Representative images of 2D gels stained with Sypro Ruby are shown (*panels e, f*). Selected protein spots that were oxidized were submitted to MALDI-TOF-MS/MS analysis for protein identification (listed in Table 1).

We employed Western blotting in conjunction with 1D-GE and 2D-GE to identify cardiac proteins that are susceptible to oxidative modifications. We incubated heart homogenates from normal rodents with 4-HNE or H_2_O_2_, and first performed 1D-GE/immunoblotting with anti-4-HNE (Fig. 3A.a) and anti-DNP (Fig. 3A.b) antibodies to detect oxidative adducts. Densitometry analysis of the Western blots showed a 25% and 35% increase in oxidation level of proteins (marked by asterisk) when cell homogenates were treated with 4-HNE and H_2_O_2_, respectively (Fig. 3A.e). To facilitate the identification of proteins susceptible to oxidative stress in humans, 4-HNE- and H_2_O_2_-treated cardiomyocyte homogenates were resolved by 2D-GE, and immunoblotting was performed to detect 4-HNE (Fig. 3B.a–b) and DNP-derivatized carbonyl (Fig. 3B.c–d) adducts. Gel images were normalized, and proteins that exhibited at least 2.5-fold increase in oxidative adducts were identified. These data showed 21 and 19 protein spots were oxidatively modified in 4-HNE-treated (Fig. 3B.b) and H_2_O_2_-treated (Fig. 3B.d) cardiomyocytes, respectively.

### Identification of cardiac proteins recognized by chagasic immune sera

**Figure 4:**
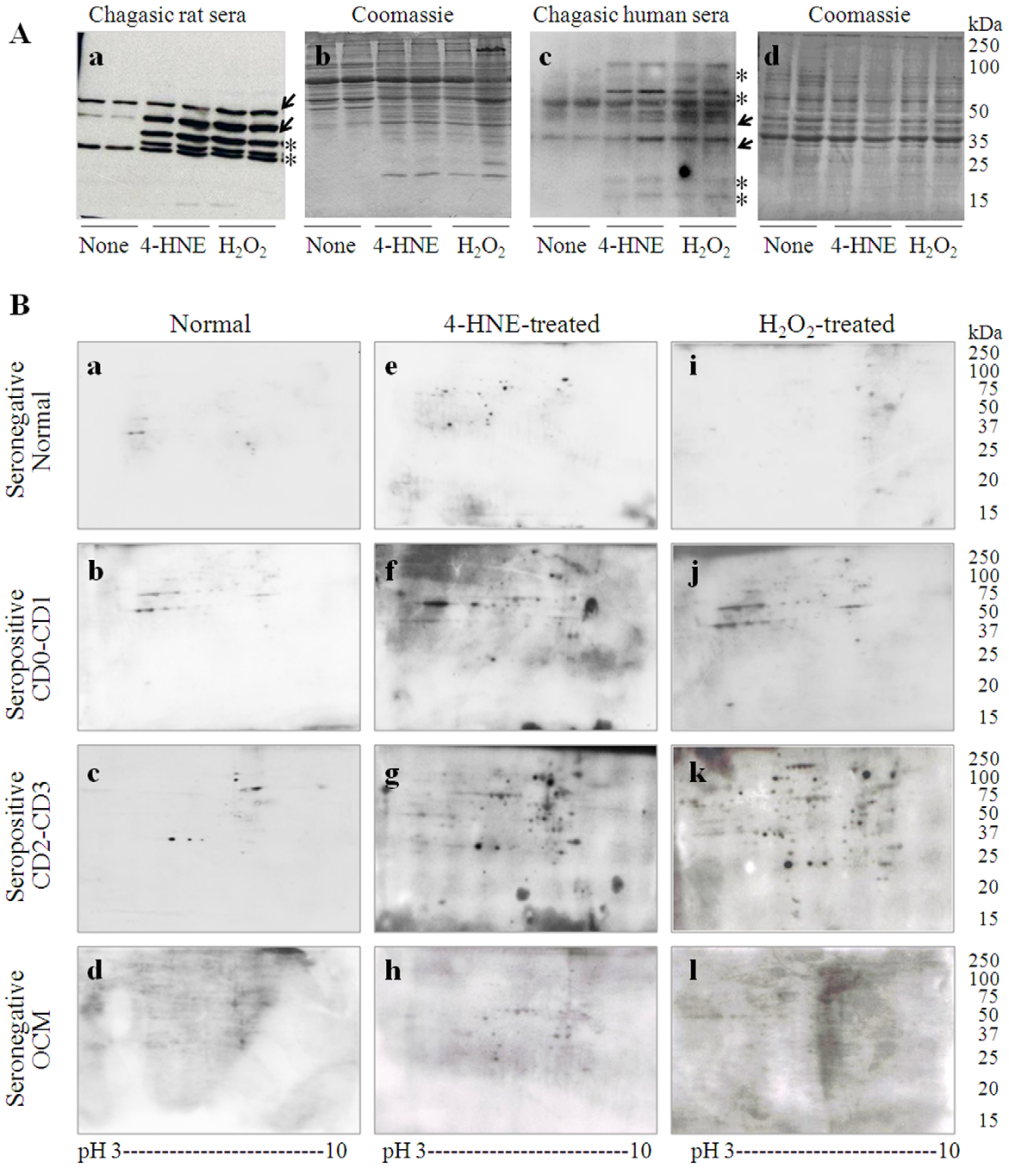
Oxidation of cardiomyocyte proteins enhanced immune recognition by antibodies in chagasic sera. (**A**) Homogenates of rat heart (*panels a, b*) and human cardiomyocytes (*panels c, d*), *in vitro* oxidized with 30 µM 4-HNE or 100 µM H_2_O_2_, were resolved by 1D-GE, and Western blotting was performed with sera from chronically infected rats (*panel a*) and humans (*panel c*). Asterisks (*panels a, c*) indicate the protein bands that were newly recognized when 4-HNE and H_2_O_2_-treated homogenates were probed with chagasic sera from chronically infected rats and humans. Arrows (*panels a, c*) indicate the protein bands that were recognized by chagasic sera at a higher intensity when 4-HNE and H_2_O_2_-treated homogenates (instead of normal/untreated homogenate) were used as antigen source, and for which densitometry was performed. Coomassie blue staining of membranes (*panels b, d*) confirmed the equal loading of samples. (**B**) Homogenates of normal cardiomyocytes (*panels a–d*) and cardiomyocytes *in vitro* oxidized with 4-HNE (*panels e–h*) or H_2_O_2_ (*panels i–l*) were resolved by 2D-GE. Western blotting was performed with sera from normal healthy controls (*panels a, e, i*), seropositive chagasic patients in the CD0–CD1 (*panels b, f, j*) or CD2–CD3 phase (*panels c, g, k*), and seronegative cardiomyopathy patients of other etiologies (*panels d, h, l*).

To determine if oxidation of cardiac proteins increased their immune recognition by sera antibodies in chagasic host, we resolved 4-HNE- and H_2_O_2_- treated rat heart (Fig. 4A) and human cardiomyocyte homogenates by 1D-GE and performed Western blotting with immune sera from chronically infected rats (Fig. 4A.a) and chagasic human patients (Fig. 4A.c). These data clearly showed that several cardiac proteins were newly recognized (marked by asterisk) and other proteins were recognized at a higher intensity (marked by arrow) by the immune sera from chagasic rats and humans when the heart homogenates treated with 4-HNE or H_2_O_2_ were used as antigen source as compared to that noted when normal cardiac homogenates were probed with chagasic sera. A densitometry analysis of the Western blots showed a 3-fold and 4-fold increase in antibody recognition of cardiac proteins when rat heart homogenates treated with 4-HNE and H_2_O_2_, respectively, were used as antigen source (Fig. 4A.a), and by 2-fold when human cardiomyocytes treated with 4-HNE or H_2_O_2_ were used as an antigen source (Fig. 4A.c). Coomassie staining of the membranes (Fig. 4A.b&d) confirmed equal loading of the samples.

Towards discovery of cardiac proteins that became immunogenic due to oxidative stress-induced modifications in human chagasic patients, homogenates of normal cardiomyocytes and cardiomyocytes *in vitro* oxidized with 4-HNE or H_2_O_2_ were resolved by 2D-gel electrophoresis, and membranes were probed with sera from clinically characterized chagasic patients (Fig. 4B). Protein spots that were differentially recognized (marked on Fig.S1) were selected for sequencing. The corresponding gels stained with Sypro Ruby (Fig.S1) were utilized to submit protein spots recognized by immune sera for MALDI-TOF-MS/MS analysis and protein identification. In total, we identified 25, 32, 76, and 40 protein spots recognized by sera IgGs from normal controls (Fig. 4B.a,e,i), chagasic patients characterized as clinically asymptomatic (CD0–CD1) (Fig. 4B.b,f,j) and symptomatic (CD2–CD3) (Fig. 4B.c,g,k), and non-chagasic/other cardiomyopathy (OCM) patients (Fig. 4B.d,h,l). In general, when we probed normal cardiomyocytes (Fig. 4B.a–d), antibody recognition was limited to 6, 9, 11, and 8 proteins by sera IgGs from normal subjects and CD0–CD1, CD2–CD3 and OCM patients, respectively. When we probed 4-HNE-oxidized cardiomyocytes (Fig. 4B.e–h), we found 21, 22, 42 and 19 protein spots were recognized by sera IgGs from normal controls and CD0–CD1, CD2–CD3 and OCM patients, respectively. When we probed H_2_O_2_-oxidized cardiomyocytes (Fig. 4B.i–l), we found 12, 30, 40 and 20 protein spots were recognized by sera IgGs from normal controls and CD0–CD1, CD2–CD3 and OCM patients, respectively. MALDI-TOF-MS/MS analysis of 82 protein spots that were uniquely oxidized and recognized by immune sera by ≥5 fold as compared to normal controls (*p*_ANOVA_≤0.01) yielded 48 unique protein identifications (Table 1). Of note is the immune recognition by chagasic sera of actin, desmin, and titin that play an important role in the assembly and function of vertebrate striated muscles, and vimentin that is a cytoskeletal component responsible for maintaining cell integrity (Table 1).

**Table 1:** Identification of proteins recognized by immune sera from chagasic patients.

Spot #	Protein name	Gene name	Accession no	Score	MW (kDa)	E value	Sera type		
							Nor	CD0–1	CD2–3
	**No treatment**								
1	Prohibitin variant	PHB	gi|62897923	95	29.8	6.29E-05	+	−	−
2	Alpha enolase like 1	ENO1	gi|3282243	428	29.8	3.15E-38	−	+++	−
3	Albumin	ALB	gi|168988718	200	67.7	1.99E-15	−	+++	−
4	Desmin	DES	gi|19908424	594	53.5	1.26E-54	−	+	−
5	Actin beta	ACTB	gi|14250401	400	41.3	1.99E-35	−	++	−
6	Aldehyde dehydrogenase, mt	ALDH	gi|6137677	544	54.3	7.92E-50	−	−	+
7	Heat shock 8 isoform 1	HSPA8	gi|5729877	989	71	2.51E-94	−	−	++
8	Heat shock protein 1 variant 1, mt	HSP1B	gi|189502784	473	60.8	9.98E-43	−	−	+++
9	Thiol antioxidant/Peroxiredoxin 2	PRX2	gi|438069	111	22	1.58E-06	−	−	+
10	Tubulin beta	TUBB	gi|57209813	538	48.1	3.15E-49	−	−	++
11	Heat shock protein 1, beta	HSP1B	gi|20149594	392	83.5	1.26E-34	−	−	−
12	Annexin A3	ANXA3	gi|4826643	85	36.5	6.29E-04	−	−	−
	**H_2_O_2_-treated**								
13**^#^**	F1ATP synthase complex alpha	ATP5A1	gi|127798841	175	59.7	6.29E-13	++	−	−
14	Annexin A2 isoform 1	ANXA2	gi|50845388	108	40.6	3.15E-06	++	−	+
15	Lamin A/C, variant 1	LMNA	gi|57014045	263	55	9.98E-22	+	+	+++
16**^#^**	Aldolase A	ALDA	gi|28595	66	11.9	5.00E-02	−	++	−
17	Annexin I, chain A	ANXA1	gi|157829895	80	35.2	1.99E-03	−	++	−
18	Guanine binding protein, beta like	GNB2L1	gi|119574080	84	30.9	7.92E-04	−	−	+++
19	Acute morphine dependence 2	CCT6A	gi|14517632	98	58.4	3.15E-05	−	−	++
20	Nucleophosmin 1 isoform 3	NPM1	gi|83641870	66	28.4	5.00E-02	−	+	++
21	Rho GDP dissociation inhibitor α	ARHGDIA	gi|4757768	84	23.2	7.92E-04	−	−	++
22	Annexin A2 isoform 1	ANXA2	gi|50845388	442	40.6	1.26E-39	+	−	+
23	Karyopherin Beta 2 like		gi|5107637	77	24.5	3.97E-03	−	−	+
24**^#^**	Enolase 1	ENO1	gi|203282367	111	47.3	1.58E-06	+	−	−
25	Heat shock protein 5	HSPA5	gi|16507237	234	72.4	9.98E-21	−	−	−
	**4-hydroxynonenal (4-HNE)-treated**								
26	Actin beta	ACTB	gi|14250401	164	41.3	7.92E-12	−	−	++
27	Heat shock protein 8 isoform 1	HSPA8	gi|5729877	524	71	7.92E-48	+	−	−
28	Prolyl 4-hydroxylase, beta subunit	P4HB	gi|20070125	491	51.6	1.58E-44	−	+	+
29*	Vimentin	VIM	gi|5030431	292	41.6	1.26E-24	−	++	−
30*	Actin beta	ACTB	gi|14250401	257	41.3	3.97E-21	−	−	+
31*	Guanine binding protein, beta	GNB2L1	gi|119574080	165	30.9	6.29E-12	++	+	+
32	Glutamate dehydrogenase	GDH	gi|20151189	354	56.3	7.92E-31	−	+++	+
33	Fascin homolog 1	FSCN1	gi|119607750	160	49.1	1.99E-11	+	−	−
34	Aldehyde dehydrogenase, mt	ALDH	gi|6137677	326	54.3	5.00E-28	+	−	−
35	Enolase 1	ENO1	gi|203282367	370	67.7	1.99E-32	−	+	++
36	Phosphopyruvate hydratase alpha	ENO1	gi|693933	236	47.3	6.29E-19	++	−	−
37	Enolase 3	ENO3	gi|153267427	142	47.2	1.26E-09	−	−	−
38	Heat shock protein 5	HSPA5	gi|16507237	723	72.4	9.98E-68	−	−	++
39	Desmin	DES	gi|55749932	509	53.5	2.51E-46	−	−	+++
40*	Laminin receptor	LRP	gi|161761214	179	53.6	2.51E-13	−	−	−
41*	Tropomyosin 1 alpha isoform 3	TPM1	gi|63252896	422	32.7	1.26E-37	+	−	−
42*	Albumin	ALB	gi|168988718	208	67.7	3.15E-16	−	++	−
43	Tryptophan 5 monooxygenase	YWHAE	gi|5803225	79	29.3	2.51E-03	+++	+	−
44	Pyruvate kinase	PKM2	gi|31416989	472	58.5	1.26E-42	−	+	++
45	Ribonucleoprotein A2, B1 isoform	RNPA2B1	gi|14043072	397	37.4	3.97E-35	−	−	+++
46	Annexin A2	ANXA2	gi|56967118	571	36.6	1.58E-52	−	++	−
47	Vimentin variant 3	VIM	gi|167887751	438	53.6	3.15E-39	−	+	+++
48*	Titin isoform novex-2	TTN	gi|110349717	51	3032.8	3.97E-21	−	+	++

Human cardiomyocyte lysates were treated with 100-µM H_2_O_2_ or 30-µM 4-hydroxynonenal (4-HNE). Cell lysates were resolved by 2 dimensional gel electrophoresis and probed with sera samples from normal subjects, seropositive chagasic patients that exhibited no clinical disease (CD0–1) or mild-moderate symptoms of clinical disease (CD2–3), and seronegative other cardiomyopathy (OCM) patients. Protein spots recognized by immune sera were submitted to MALDI-TOF-MS/MS analysis. A −, +, ++, and +++ indicates none, mild, moderate and strong recognition of protein spots by immune sera. Gels were also probed with anti-dinitrophenyl (DNP) and anti-4-HNE antibodies. In column 1, * and ^#^ indicate protein spots that were recognized by anti-HNE and anti-DNP antibodies, respectively.

### Validation of antigenicity of oxidized cardiac proteins in chagasic patients

**Figure 5:**
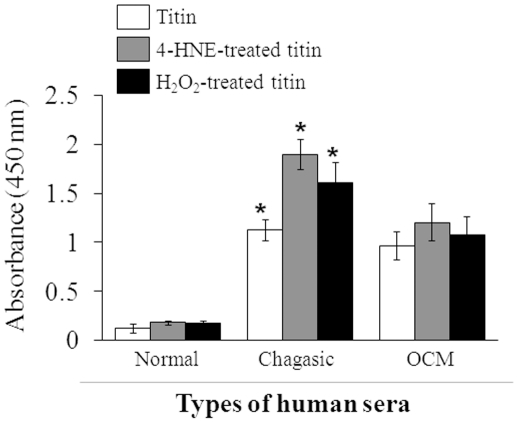
Oxidized titin exhibited increased immunogenicity in chagasic patients. Recombinant titin was *in vitro* oxidized with 4-HNE (30 µM) or H_2_O_2_ (100 µM). An enzyme-linked immune-sorbent assay (ELISA) was performed to titer the antibody level against recombinant and oxidized titin in sera samples of normal healthy controls, seropositive chagasic patients, and seronegative subjects with cardiomyopathy of other etiologies (OCM). n = 25/group (*p<0.05).

To confirm that oxidation-induced changes indeed increased the immunogenicity of cardiac proteins in chagasic patients, we utilized human recombinant titin protein (0.1 mg/ml), *in vitro* oxidized with 30 µM 4-HNE or 100 µM H_2_O_2_, and performed an ELISA to identify the serum levels of anti-titin antibodies in chagasic patients (Fig. 5). We detected moderate levels of anti-titin antibodies in the sera of chagasic patients compared to that in normal controls. The antibody recognition of titin by sera antibodies of chagasic patients was increased by 50–70% when titin oxidized with 4-HNE or H_2_O_2_ was used as an antigen. The detection of anti-titin antibodies in sera from chagasic patients correlated directly with the disease stage, with a moderate increase in patients at the CD0/CD1 stage (data not shown), while a substantial increase was noted in patients at the CD2/CD3 stage (Fig. 5). In comparison, seronegative patients with cardiac symptoms due to other etiologies (OCM) exhibited a moderate level of anti-titin antibodies. The immune recognition of oxidized titin was not increased in OCM patients (Fig. 5). These data validated the experimental results presented in Fig. 4 and suggested to us that the oxidative stress-induced changes in cardiac antigens enhanced their immunogenicity and recognition by antibodies elicited in human chagasic patients.

### Pathologic significance of immune recognition of oxidized cardiac antigens in Chagas disease

**Figure 6:**
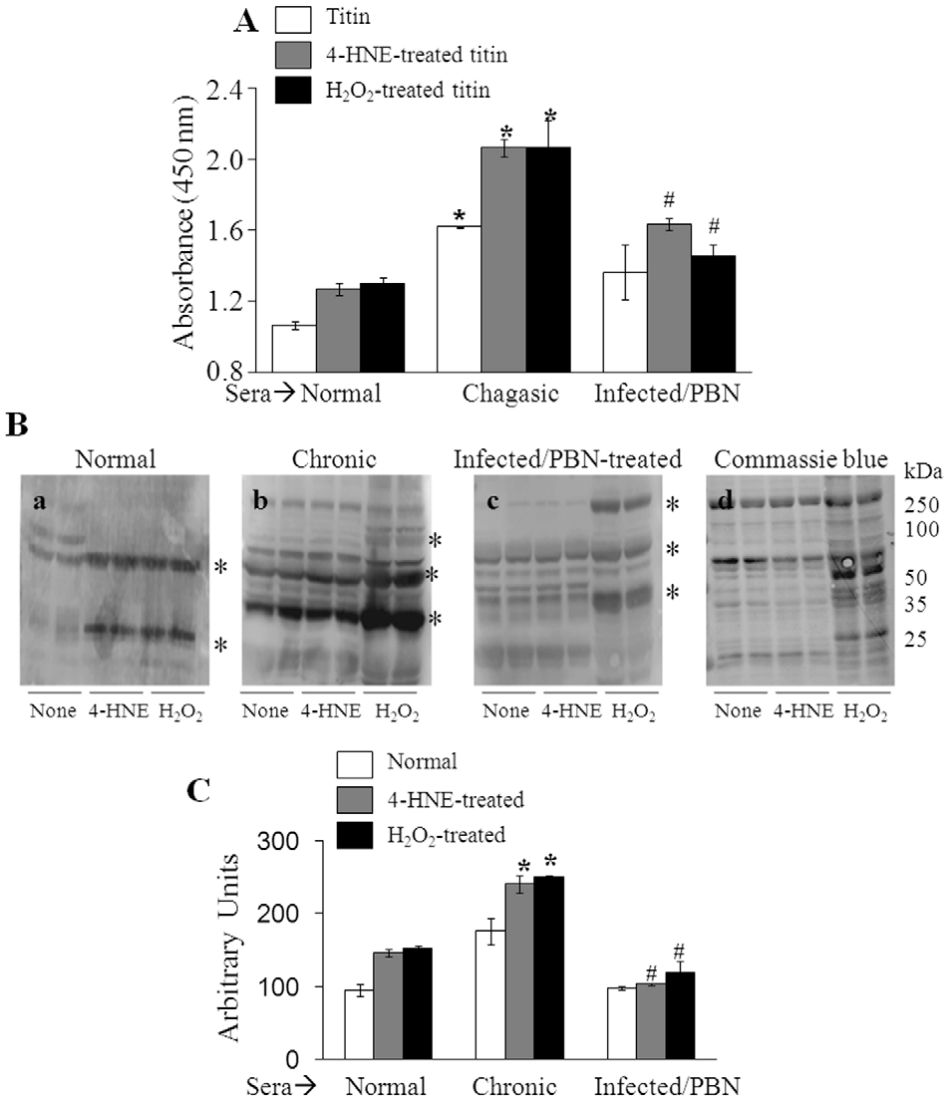
Antioxidant treatment arrested the immune recognition of oxidized cardiac antigens in chagasic rats. Sprague Dawley rats were infected with *T. cruzi* and treated with phenyl-α-tert-butyl nitrone (PBN, antioxidant) as detailed in Materials and Methods. (**A**) Recombinant titin was *in vitro* oxidized with 4-HNE (30 µM) or H_2_O_2_ (100 µM). An ELISA was performed to titer the antibody levels against recombinant and oxidized titin in sera of normal rats, and rats that were chronically infected and treated with PBN. (**B**) Rat heart homogenates (normal and *in vitro* oxidized with 4-HNE or H_2_O_2_) were resolved by 1D-GE and Western blotting was performed with sera from normal *(panel a)*, chronically infected *(panel b)* and infected/PBN-treated *(panel c)* rats. Coomassie blue staining of membranes *(panel d)* confirmed the equal loading of samples. n = 6/group (*p<0.05). (**C**) Densitometry analysis of protein bands (marked by asterisks in **B**).

To corroborate the pathological significance of oxidized cardiac antigens in Chagas disease, we treated the infected rats with PBN to control oxidative stress [24]. We collected immune sera of infected (with and without treatment) rats during the acute and chronic stages of infection and disease development, and utilized them to determine the levels of anti-titin antibodies by an ELISA that used recombinant and oxidized titin as antigens (Fig. 6A). As expected from the results presented in Fig. 5, our data clearly demonstrated that an increased detection of recombinant titin by sera antibodies in infected/untreated chagasic rats was further enhanced when titin was oxidized with 4-HNE or H_2_O_2_. It is important to note that PBN-mediated control of cardiac pathology and preservation of heart contractile function in chagasic rats [24] were associated with an up to 50% decline in serum levels of antibodies to cardiac titin (normal and oxidized forms) in chronically infected/treated rats (Fig. 6A). Likewise, rat heart homogenates, *in vitro* oxidized with 4-HNE or H_2_O_2_, as compared to normal rat homogenate, exhibited 53% and 60%, respectively, increase in immune recognition by chagasic sera antibodies (Fig. 6B.b & C). The immune recognition of cardiac proteins was significantly controlled when rat heart homogenates (normal and *in vitro* oxidized with 4-HNE or H_2_O_2_) were submitted to Western blotting with sera samples from infected/PBN-treated rats, compared to that found with sera samples from infected/untreated rats (Fig. 6B.c & C). Together, these data demonstrated that the antigenicity of cardiac proteins is enhanced in an oxidative stress-dependent manner in chagasic rats. The control of antibody responses against cardiac proteins (normal and oxidized) in infected/PBN-treated rats that also exhibited a gain of cardiac function [24] suggested to us that anti-cardiac antibodies are of pathologic importance in Chagas disease development.

## Discussion

In this study, we examined the pathological significance of oxidative stress in human chagasic patients. We demonstrated that myocardial levels of oxidative modifications (4-HNE, carbonyls, MDA, and 3-NT adducts) were significantly increased in chagasic patients and experimental animals, and affected cardiomyocytes viability. By 2D-GE/WB, we found that 48 cardiac proteins (native or oxidized) exhibited a significant increase in immune recognition by antibodies in chagasic sera and were identified by MALDI-TOF-MS/MS analysis. Molecular and functional analysis allocated a majority of the cardiac proteins recognized by chagasic sera to the cell death/cell proliferation category and to molecular pathways associated with hypoxia, mitochondrial swelling and permeability transition, and hypertrophy and dilation of the left ventricle. The 2D-GE/WB results were validated by an ELISA demonstrating recombinant titin, when *in vitro* oxidized, was highly immunogenic, and the target of serum antibodies in chagasic patients and experimental animals. Importantly, treatment of infected rodents with PBN antioxidant resulted in an up to 50% decline in serum levels of antibodies to cardiac-oxidized proteins, associated with control of cardiac pathology and preservation of heart contractile function in chagasic rats [23], [24]. To the best of our knowledge, this is the first study demonstrating that oxidative stress-induced modifications of cardiac proteins results in enhanced immune recognition by serum antibodies in chagasic patients, and this response parallels with the pathologic events during Chagas disease development.

The etiology of Chagas disease pathogenesis is complex. The presence of parasite-specific immune responses to *T. cruzi* parasites or antigens persistent in the heart has been considered the most accepted hypothesis to explain inflammatory pathology [29], [30] and resultant myocytolysis in chagasic hearts. Yet, the persistence and extent of inflammatory processes in chagasic hearts are not in sync with the low burden of parasites in the chronic stage, thus, suggesting to us that host responses contribute to the risk of Chagas disease development. Accordingly, others have attributed antibody-mediated cytotoxicity [31] and damage caused by tissue-infiltrating neutrophils [32] to induction of autoimmunity against cardiac antigens [33], [34] in chagasic patients. We and others have previously shown that oxidative/nitrosative stress is increased during the course of *T. cruzi* infection and disease development in experimental animals and chagasic patients (reviewed in [4]). Our current studies suggest that exacerbated oxidative stress induced protein modifications provide epitopes to drive the self-directed antibody responses in chagasic patients. Other studies have demonstrated that ROS elicit inflammatory cytokines (e.g., TNF-α, IFN-γ, IL-1β) in cardiomyocytes infected by *T. cruzi* [35], [36]. Inflammatory pathology was controlled in chronically infected experimental animals and human patients by enhancing the antioxidant status, which was also beneficial in preserving the cardiac function during Chagas disease [23], [24], [37], [38]. Our recent observations indicate that the mitochondrial release of ROS due to electron transport chain dysfunction and enhanced release of electrons to molecular oxygen is the primary source of oxidative stress in the heart [10]. These results allow us to propose a unified hypothesis of the pathogenesis of Chagas disease. We surmise that ROS are initiated by *T. cruzi* via activation of inflammatory responses (NADPH oxidase) [39] during the acute stage, and consistently produced due to mitochondrial dysfunction during the chronic stage [10]. These ROS, via their ability to modify DNA, protein and lipids, cause myocytolysis and damage to cardiac microvasculature. At the same time, oxidized proteins serve as neoantigens, thereby providing a stimulus for the activation of innate (neutrophils, macrophages) and antibody response and the resultant immunocytotoxicity. Our hypothesis is supported by the observations that treatment with PBN of infected rodents provided a significant control of cardiac oxidative pathology, cardiac remodeling, and antibody cytotoxicity directed against cardiac proteins, and consequently was beneficial in preserving cardiac hemodynamics and contractile function [23], [24].

It is important to note that benefits afforded by PBN treatment were not linked to diminished persistence of parasites. We have found similar or higher levels of *T. cruzi*, determined by real time PCR approach, in chronically infected/PBN-treated rats as compared to chronically infected/untreated chagasic rats [23], [24]. This observation provide further support to the idea that parasite persistence is not sufficient to sustain the chronic evolution of disease, and host factors (e.g., oxidative stress, oxidized antigens) play an important role in determining the disease outcome.

Ingenuity Pathway Analysis (IPA) is a highly curated and comprehensive software used for the integration of proteins into networks and pathways with biological meaning [40]. Molecular analysis of the cardiac proteins that were oxidized and/or targets of antibodies in chagasic sera suggested to us that 19 of the 48 identified proteins (ACTB, ALDOA, ARHGDIA, ATP5A1, CCT6A, ENO1, GNB2L1, HSP90AB1, HSPA5, HSPA8, HSPD1, NPM1, P4HB, PHB, PKM2, PRDX2, TPM1, VIM, YWHAE, p-value: 1.07E-09) play an important role in regulating cell death and cell proliferation. Following functional analysis, it was clear that a majority of identified proteins were associated with a decrease in (ALDOA, ARHGDIA, ATP5A1, CCT6A, GNB2L1, HSP90AB1, HSPA5, HSPA8, P4HB, PHB, and YWHAE) or regulation of (ENO1, HSPD1, NPM1, and PRDX2) cell death [41]. Many of the proteins involved in cell death regulation (i.e., ATP5A1, GNB2L1, HSPA5, HSPD1, NPM1, and PRDX2) were also relevant with respect to cell growth and proliferation [42], [43], [44]. Other proteins identified in this study (ANXA2, DES, FSCN1, LMNA, PKM2ENO1 and STAT5B) have also been characterized to regulate cell growth and proliferation [42], [43], [44]. Only a few proteins, i.e., EGCG, HNRNPA2B1, and TPM1, have been characterized to induce cell death and/or decrease cell growth in response to various stimuli [45]. These analyses suggested that cardiomyocytes proteins that play a role in cell survival and/or cell proliferation were disproportionately targeted by oxidative stress and antibody response in chagasic conditions. Indeed, myocardial necrosis [46] associated with a compromised regeneration of cardiomyocytes [47] are often observed in clinically symptomatic chagasic experimental animals and human patients. IPA analysis results led us to suggest that EGCG, FSCN1, LMNA, PRDX2, and NPM1 affect, while ANXA2, DES, ENO1, HSPD1, LMNA, VIM, and MYOD1 regulate differentiation of mouse myoblasts (or other cell types) in response to various stimuli. We surmise that oxidative modifications and antigenicity of the cardiac proteins involved in the regulation of cell survival and cell growth prevent the preservation of myocardial structure during Chagas disease; however, this will be verified in future studies.

It also became apparent to us that in addition to their role in cardiovascular disease, the immunome signature of chagasic patients (ANXA2, ARHGDIA, FSCN1, GNB2L1, HSP90AB1, HSPA5, HSPA8, P4HB, PKM2, VIM, YWHAE, p-value: 1.37E-09) was also indicative of gastrointestinal disorder. Megacolon, by some estimates, is an under-reported clinical outcome in chagasic patients [48]. Our studies do not delineate the cellular or tissue origin of the oxidized proteins, as we have utilized an *in vitro* model to test our hypothesis of significance of oxidative stress in eliciting neo-antigens. Irrespective of the source of oxidized proteins, it can be deduced that antibodies, once formed, will not be restricted to the myocardium, and can bind to and alter proteins in multiple tissues and organs. Indeed, others have reported a predominance of β1-adrenergic receptor (AR) and muscarinergic 2 (M2) receptor autoantibodies in Chagas cardiomyopathy patients and of β2-AR with M2 autoantibodies in megacolon patients, and have suggested their utility for risk assessment of clinical disease [49]. We surmise that the oxidative stress-induced formation of oxidized proteins/antigens also has implications in the development of gastrointestinal disorders in chagasic patients and that organ selectivity of pathology may be determined by the tissue preference of the invading parasite strain and resultant cellular source of target (oxidized) antigens.

In summary, we have provided the first evidence that oxidative stress-induced protein modifications enhanced a self-directed antibody response in chagasic experimental animals and human patients. The oxidation and antigenicity of several of the proteins that are required for regulating cell death and cell proliferation can adversely impact myocardial structure during Chagas disease. Further, we show that the antigenicity of the oxidized self-proteins may also be of pathological significance in the development of gastrointestinal disorders in chagasic patients. Our data provide us with an impetus to investigate the potential utility of ROS scavengers as adjunct therapy in arresting the progression of Chagas disease.

## Supporting Information

Figure S1 **Identification of cardiac protein spots recognized by antibodies in chagasic sera.** Homogenates of normal cardiomyocytes (*panels a–d*) and cardiomyocytes *in vitro* oxidized with 4-HNE (*panels e–h*) or H_2_O_2_ (*panels i–l*) were resolved by 2D-GE. Western blotting was performed with sera from normal healthy controls (*panels a,e,i*), seropositive chagasic patients in CD0–CD1 phase (*panels b,f,j*) or CD2–CD3 phase (*panels c,g,k*), and seronegative cardiomyopathy patients of other etiologies (*panels d,h,l*). Western blots were digitalized on a ProXPRESS Proteomic Imaging System (Perkin Elmer), and the images were analyzed on Progenesis SameSpotst™ software 2.0 (NonLinear Dynamics). Normalized spot volumes, i.e., the volume of each spot over the volume of all spots in the gel, were used for comparison of the different groups, and candidaes were identified as protein spots that changed at least 5-fold versus their specific control. Candidate protein spots were cut from the corresponding Sypro Ruby-stained gels, and submitted for MALDI-TOF-MS/MS analysis for protein identification (listed in Table 1).
(DOCX)
