# Supplementary material for: Cardiac-Oxidized Antigens Are Targets of Immune Recognition by Antibodies and Potential Molecular Determinants in Chagas Disease Pathogenesis
Source: PLoS One. 2012 Jan 4;7(1):e28449. doi: 10.1371/journal.pone.0028449 (PMC3251564; doi:10.1371/journal.pone.0028449)
Supplement: Figure S1 — Identification of cardiac protein spots recognized by antibodies in chagasic sera. Homogenates of normal cardiomyocytes (panels a–d) and cardiomyocytes in vitro oxidized with 4-HNE (panels e–h) or H2O2 (panels i–l) were resolved by 2D-GE. Western blotting was performed with sera from normal healthy controls (panels a,e,i), seropositive chagasic patients in CD0–CD1 phase (panels b,f,j) or CD2–CD3 phase (panels c,g,k), and seronegative cardiomyopathy patients of other etiologies (panels d,h,l). Western blots were digitalized on a ProXPRESS Proteomic Imaging System (Perkin Elmer), and the images were analyzed on Progenesis SameSpotst™ software 2.0 (NonLinear Dynamics). Normalized spot volumes, i.e., the volume of each spot over the volume of all spots in the gel, were used for comparison of the different groups, and candidaes were identified as protein spots that changed at least 5-fold versus their specific control. Candidate protein spots were cut from the corresponding Sypro Ruby-stained gels, and submitted for MALDI-TOF-MS/MS analysis for protein identification (listed in Table 1). (DOCX) [file pone.0028449.s001.docx]

**Figure S1**

**Cardiac Oxidized Antigens Are Targets of Immune Recognition by Antibodies and Potential Molecular Determinants in Chagas Disease Pathogenesis**

Monisha Dhiman, PhD^1^, Paola M. Zago, PhD^2^, Sonia Nunez, MD^3^, Federico Nunez-Burgio^3^, MD, and Nisha Jain Garg, PhD^1 ,4*^

^1^ Department of Microbiology and Immunology, ^4^Center for Tropical Diseases, and Institute for Human Infections and Immunity, The University of Texas Medical Branch, Galveston, TX

^2^ Instituto de Patología Experimental, Facultad de Ciencias de la Salud, Universidad Nacional de Salta, Salta, Argentina

^3^ Hospital Público de Gestión Descentralizada San Bernardo, Salta, Argentina

**Figure S1. Identification of cardiac protein spots recognized by antibodies in chagasic sera.** Homogenates of normal cardiomyocytes (*panels a-d*) and cardiomyocytes *in vitro* oxidized with 4-HNE (*panels e-h*) or H_2_O_2_ (*panels i-l*) were resolved by 2D-GE. Western blotting was performed with sera from normal healthy controls (*panels a,e,i*), seropositive chagasic patients in CD0-CD1 phase (*panels b,f,j*) or CD2-CD3 phase (*panels c,g,k*), and seronegative cardiomyopathy patients of other etiologies (*panels d,h,l*). Western blots were digitalized on a ProXPRESS Proteomic Imaging System (Perkin Elmer), and the images were analyzed on Progenesis SameSpotst™ software 2.0 (NonLinear Dynamics). Normalized spot volumes, i.e., the volume of each spot over the volume of all spots in the gel, were used for comparison of the different groups, and candidaes were identified as protein spots that changed at least 5-fold versus their specific control. Candidate protein spots were cut from the corresponding Sypro Ruby-stained gels, and submitted for MALDI-TOF-MS/MS analysis for protein identification (listed in Table 1).
